# ECDC’s latest publications

**DOI:** 10.2807/1560-7917.ES.2017.22.50.171214-4

**Published:** 2017-12-14

**Authors:** Eurosurveillance editorial team

**Affiliations:** 1 European Centre for Disease Prevention and Control (ECDC), Stockholm, Sweden

**Keywords:** infectious diseases, public health, Europe

**Rapid risk and outbreak assessments**

Multi-country outbreak of *Salmonella* Enteritidis infections linked to Polish eggs

Date of publication: 12 December 2017

https://ecdc.europa.eu/en/publications-data/multi-country-outbreak-salmonella-enteritidis-infections-linked-polish-eggs

Multi-country outbreak of *Listeria* *monocytogenes* PCR serogroup IVb, MLST ST6

Date of publication: 8 December 2017

https://ecdc.europa.eu/en/publications-data/multi-country-outbreak-listeria-monocytogenes-pcr-serogroup-ivb-mlst-st6

Outbreak of travel-associated Legionnaires' disease - Palmanova, Mallorca (Spain), September - October 2017

Date of publication: 23 October 2017

https://ecdc.europa.eu/en/publications-data/outbreak-travel-associated-legionnaires-disease-palmanova-mallorca-spain

Outbreak of pneumonic plague in Madagascar: recent introduction in the Seychelles

Date of publication: 13 October 2017

https://ecdc.europa.eu/en/publications-data/rapid-risk-assessment-outbreak-pneumonic-plague-madagascar-recent-introduction

Outbreak of plague in Madagascar, 2017 

Date of publication: 9 October 2017

https://ecdc.europa.eu/en/publications-data/rapid-risk-assessment-outbreak-plague-madagascar-2017

Clusters of autochthonous chikungunya cases in Italy 

Date of publication: 9 October 2017

https://ecdc.europa.eu/en/publications-data/rapid-risk-assessment-clusters-autochthonous-chikungunya-cases-italy-first-update

**Technical reports**

Guide to revision of national pandemic influenza preparedness plans: Lessons learned from the 2009 A(H1N1) pandemic

Date of publication: 23 November 2017

https://ecdc.europa.eu/en/publications-data/systematic-review-active-case-finding-communicable-diseases-prison-settings

Training needs assessment for EU/EEA countries: Assessment methodology and 2015 survey 

Date of publication: 26 October 2017

https://ecdc.europa.eu/en/publications-data/training-needs-assessment-eueea-countries-assessment-methodology-and-2015-survey

ECDC, EFSA and EMA Joint Scientific Opinion on a list of outcome indicators as regards surveillance of antimicrobial resistance and antimicrobial consumption in humans and food-producing animals 

Date of publication: 26 October 2017

https://ecdc.europa.eu/en/publications-data/ecdc-efsa-and-ema-joint-scientific-opinion-list-outcome-indicators-regards

Vector control with a focus on *Aedes* *aegypti* and *Aedes* *albopictus* mosquitoes - Literature review and analysis 

Date of publication: 16 October 2017

https://ecdc.europa.eu/en/publications-data/vector-control-focus-aedes-aegypti-and-aedes-albopictus-mosquitoes-literature

Public health emergency preparedness: Core competencies for EU Member States 

Date of publication: 5 October 2017

https://ecdc.europa.eu/en/publications-data/public-health-emergency-preparedness-core-competencies-eu-member-states

**Scientific advice**

Expert opinion on the introduction of the meningococcal B (4CMenB) vaccine in the EU/EEA

Date of publication: 6 December 2017

https://ecdc.europa.eu/en/publications-data/expert-opinion-introduction-meningococcal-b-4cmenb-vaccine-eueea

Systematic review on active case finding of communicable diseases in prison settings

Date of publication: 23 November 2017

https://ecdc.europa.eu/en/publications-data/systematic-review-active-case-finding-communicable-diseases-prison-settings

**Technical documents**

Effective use of digital platforms for HIV prevention among men who have sex with men in the European Union/European Economic

Date of publication: 1 December 2017

https://ecdc.europa.eu/en/publications-data/effective-use-digital-platforms-hiv-prevention-among-men-who-have-sex-men

Guidance for the management of suspected bubonic plague cases identified on aircraft and ships

Date of publication: 30 October 2017

https://ecdc.europa.eu/en/publications-data/guidance-management-suspected-bubonic-plague-cases-identified-aircraft-and-ships

Information for travellers to Madagascar 

Date of publication: 27 October 2017

https://ecdc.europa.eu/en/publications-data/information-travellers-madagascar

Guidance for healthcare workers on the use of personal protective equipment in the management of bubonic and pneumonic plague patients

Date of publication: 26 October 2017

https://ecdc.europa.eu/en/publications-data/guidance-healthcare-workers-use-personal-protective-equipment-management-bubonic

Guidance for the management of suspected pneumonic plague cases identified on aircraft and ships

Date of publication: 26 October 2017

https://ecdc.europa.eu/en/publications-data/guidance-management-suspected-pneumonic-plague-cases-identified-aircraft-and

**Surveillance reports**

The European Union summary report on trends and sources of zoonoses, zoonotic agents and food-borne outbreaks in 2016

Date of publication: 12 December 2017

https://ecdc.europa.eu/en/publications-data/european-union-summary-report-trends-and-sources-zoonoses-zoonotic-agents-and-9

Monthly measles and rubella monitoring report, December 2017

Date of publication: 08 December 2017

https://ecdc.europa.eu/en/publications-data/monthly-measles-and-rubella-monitoring-report-december-2017

HIV/AIDS surveillance in Europe 2017 – 2016 data

Date of publication: 28 November 2017

https://ecdc.europa.eu/en/publications-data/hivaids-surveillance-europe-2017-2016-data

Surveillance of antimicrobial resistance in Europe 2017

Date of publication: 15 November 2017

https://ecdc.europa.eu/en/publications-data/surveillance-antimicrobial-resistance-europe-2017

Monthly measles and rubella monitoring report, November 2017

Date of publication: 10 November 2017

https://ecdc.europa.eu/en/publications-data/monthly-measles-and-rubella-monitoring-report-november-2017

Influenza virus characterisation, Summary Europe, September 2017 

Date of publication: 23 October 2017

https://ecdc.europa.eu/en/publications-data/influenza-virus-characterisation-summary-europe-september-2017

ECDC/EFSA joint report: Avian influenza overview October 2016-August

Date of publication: 16 October 2017

https://ecdc.europa.eu/en/publications-data/ecdcefsa-joint-report-avian-influenza-overview-october-2016-august-2017

Monthly measles and rubella monitoring report, October 2017 

Date of publication: 13 October 2017

https://ecdc.europa.eu/en/publications-data/monthly-measles-and-rubella-monitoring-report-october-2017

Bi-annual measles and rubella monitoring report, October 2017

Date of publication: 13 October 2017

https://ecdc.europa.eu/en/publications-data/bi-annual-measles-and-rubella-monitoring-report-october-2017

Annual Epidemiological Report series on communicable diseases in Europe

Date of publication: released as new disease chapters become available

https://ecdc.europa.eu/en/annual-epidemiological-reports

ECDC communicable disease threats report

Date of publication: every Friday

https://ecdc.europa.eu/en/threats-and-outbreaks/reports-and-data/weekly-threats

**Corporate publication**

Evaluation of ECDC Ebola deployment in Guinea 

Date of publication: 17 October 2017

https://ecdc.europa.eu/en/publications-data/evaluation-ecdc-ebola-deployment-guinea

**Mission report**

ECDC country visit to Luxembourg to discuss antimicrobial resistance issues

Date of publication: 21 November 2017

https://ecdc.europa.eu/en/publications-data/ecdc-country-visit-luxembourg-discuss-antimicrobial-resistance-issues

**Scientific peer-reviewed publications**

Publications by ECDC staff in scientific journals

Updated monthly

https://ecdc.europa.eu/en/publications-data?f%5b0%5d=output_types%3A1247

